# Virtual care: Enhancing access or harming care?

**DOI:** 10.1177/0840470420938818

**Published:** 2020-07-20

**Authors:** Lorian Hardcastle, Ubaka Ogbogu

**Affiliations:** 1 Faculty of Law and Cumming School of Medicine, 2129 University of Calgary, Calgary, Alberta, Canada.; 2 Faculty of Law and Faculty of Pharmacy & Pharmaceutical Sciences, 3158 University of Alberta, Edmonton, Alberta, Canada.

## Abstract

COVID-19 has catalyzed the adoption of virtual medical care in Canada. Virtual care can improve access to healthcare services, particularly for those in remote locations or with health conditions that make seeing a doctor in person difficult or unsafe. However, virtual walk-in clinic models that do not connect patients with their own doctors can lead to fragmented, lower quality care. Although virtual walk-in clinics can be helpful for those who temporarily lack access to a family doctor, they should not be relied on as a long-term substitute to an established relationship with a primary care provider. Virtual care also raises significant privacy issues that policy-makers must address prior to implementing these models. Patients should be cautious of the artificial intelligence recommendations generated by some virtual care applications, which have been linked to quality of care concerns.

## Introduction

COVID-19 fast-tracked the adoption of virtual medical care in Canada. With recommendations that individuals stay home and health professionals minimize face-to-face interactions with patients, provinces looked to virtual care as a means of facilitating safe access to health services. In this article, we analyze virtual care models in Canada and argue that although they can improve access to health services, policy-makers must approach them with caution due to quality of care and privacy issues. This is particularly important as provinces transition from the virtual models adopted in response to COVID-19 to those that will be relied on as permanent features of the health delivery system.

## Models of virtual care in Canada

There are two main models of virtual care in Canada. The first facilitates communication between patients and their own physicians, while the second is a virtual walk-in clinic. Some provinces employ both models.

In response to COVID-19, provinces modified existing billing codes or created new ones to enable virtual communications within existing clinical relationships.^1,2^ Depending on the province, these billing codes allow doctors to consult with their patients via phone, an app designed specifically for medical consultations, or a general videoconferencing app like Zoom. Self-regulatory bodies or provincial governments may recommend or require the use of particular platforms to facilitate virtual visits.

Some provinces have also opted to pay for virtual walk-in clinic visits, whereby patients log into an app and are matched with a licensed physician with whom they do not have a preexisting clinical relationship. In this regard, British Columbia, Alberta, and Ontario reimburse physician visits through Babylon by Telus Health, while Saskatchewan insures Lumeca consultations.^3^ In addition to publicly funded virtual providers, there are various virtual providers that charge patients directly, such as Maple.

## Access and quality of care issues

When technology is used to support communications between patients and their own doctors, virtual options may enhance quality of care. For example, patients who have contagious illnesses or compromised immune systems can consult with physicians without the risk of transmission. Virtual options may also improve access for patients with mobility issues or those who live in remote areas. Rural access could be further enhanced if provincial colleges collaborate on telemedicine, so that licensing rules do not act as a barrier to the cross-provincial practice of medicine.^4^

Prior to COVID-19, patients expressed a desire for expanded virtual options, with a Canada Health Infoway Survey finding that while 41% of Canadians wanted virtual visits with their provider, only 4% of family doctors offered this option.^5^ However, virtual care requires access to the requisite technology and an ability to use that technology, which certain patient populations may struggle with. Furthermore, virtual care may be inappropriately distant for some encounters such as sharing a cancer diagnosis with a patient or conducting certain mental health consultations. The Canadian Medical Association lists additional symptoms that are not amenable to virtual care: ear pain, cough, abdominal/gastrointestinal symptoms, musculoskeletal issues, most neurological symptoms, and congestive heart failure.^6^

When technology is used as a virtual walk-in clinic rather than a means of communicating with one’s own doctor, quality of care may suffer. There is a significant body of literature exploring the impact of continuity of care on patient outcomes. One crucial element of continuity is an ongoing relationship with a primary care provider, particularly for patients with chronic or complex medical conditions. For example, studies link continuity of care to improved health outcomes, reductions in emergency department visits, and fewer hospitalizations.^7,8^

Continuity of care may also suffer due to limits on access to health information. Virtual walk-in clinic physicians may have access to some medical records (by virtue of being licensed in that jurisdiction and thus able to log onto the provincial electronic medical record system), but these records may not include notes made by family physicians or other reports. In addition, notes from virtual consults via Babylon are not shared with the patient’s regular doctor unless the patient requests this.^9^

Several physician groups have expressed concerns with virtual walk-in clinics. For example, the President of the Alberta Medical Association noted problems with Babylon’s focus on “episodic care.” She noted that patients access a “small number of doctors who work in rotation” rather than their own family physicians and there is “no mechanism to assign patients to a consistent Babylon physician in order to maintain continuity.”^9^ The College of Physicians and Surgeons of British Columbia has received several allegations of deficient care provided by virtual walk-in clinics, with the Inquiry Committee concluding that it is “almost impossible for physicians to meet expected standards for the majority of patients presenting with episodic concerns in this fashion.”^10^

Virtual walk-in clinics may also drive up healthcare costs and raise equity concerns. For example, a UK study found that patients used Babylon consultations “more than would be expected given their age and health status,” although the authors could not determine whether this was driven by the accessibility of the service, supply-induced demand, or unmet medical need.^11^ This study found that the vast majority of Babylon patients were younger, healthier, and lived in wealthier areas, suggesting that virtual care may facilitate inequitable access to physicians. Policy-makers should collect comprehensive Canadian data on the divide between those who access virtual care and those who see doctors in person as medical practices re-open following COVID-19, so that they can address inequities.

While some provinces insure virtual visits provided through certain apps, others require patients to pay for consultations. Maple, one popular platform, charges patients $49 for weekday visits, $79 for weekend visits, and $99 for overnight visits.^12^ This raises equity concerns because it allows wealthier people (who, on average, tend to be healthier^13^) to jump the queue and purchase quick access to medical care, which may be delivered by doctors who would otherwise spend their time treating patients in the public system according to need rather than ability to pay.

One possible benefit of the virtual walk-in clinic model is improved access to care for patients who do not have family physicians or who live in remote areas without doctors. However, if patients do not have established relationships with family physicians, these apps should not become their primary point-of-contact with the healthcare system. Instead, virtual walk-in clinics should be a temporary, stop-gap measure and governments must prioritize policies that ensure that all Canadians have an established relationship with a primary care provider. Because of these concerns, it is problematic for governments to encourage the use of this model of care as they have done in some provinces,^3^ or for Telus to send emails to its customers advertising Babylon, given that this may prompt patients who have family physicians to use more convenient, but also more fragmented, virtual options.

## Privacy concerns

Regardless of the mode of delivery, virtual care must comply with privacy laws. In most provinces and territories, the relevant laws include health information statutes and, in some provinces, privacy legislation that applies to the private sector. The federal *Personal Information and Protection of Electronic Documents Act* (PIPEDA),^14^ which governs personal information collected, used or disclosed by private-sector organizations in the course of commercial activities, can also apply. This legislation applies to personal information that crosses provincial/territorial and national borders and is especially relevant to regulating virtual care provided by out-of-province or foreign healthcare professionals and businesses. Similarly, providers operating within Canada who move health information across borders are subject to PIPEDA.

Although the impact of privacy laws on virtual care systems will become clearer as these systems mature, several privacy-related concerns have emerged. These include questions surrounding custodianship and ownership of virtual care health information, broad authorization for unspecified use and sharing of patient health information, and data security.

When patients virtually consult their own doctors, custodianship and ownership of health information is likely not an issue. Generally, persons or entities designated as custodians can collect and share health information under health information protection statutes. Physicians delivering virtual care to their existing patients are health information custodians and are bound by routine rules regarding the collection, use, and disclosure of health information. Based on applicable legal doctrine, health information collected at point of care belongs to the patient who provided it.^15^

With the virtual walk-in clinic model, more vexing considerations arise. While a physician who delivers care under this model is a custodian under the health information statute of the jurisdiction where he or she is licensed, the status of companies that operate virtual walk-in clinics is less clear. For example, Babylon has not been designated as a custodian under Alberta’s *Health Information Regulation* ^16^ or equivalent British Columbia and Ontario rules. This means that the company is potentially not bound by Alberta’s *Health Information Act*.^17^ Since these companies will inevitably have access to patient health information—Babylon’s terms of service stipulate such access—it is concerning that they may escape regulatory reach.

Rules regarding ownership of health information may be difficult to implement or enforce where virtual walk-in companies are domiciled in or operate outside a province or outside Canada. Babylon’s Privacy Policy states, for example, that patient data may be “processed, stored, or accessed” by Babylon and its service providers (both within and outside Canada) or accessed by foreign governments under applicable law. Given the implications of such widespread use and sharing of a patient’s health information, companies like Babylon ought to be clearly designated custodians under applicable provincial laws.

A related concern is the use of terms of service or click-through agreements to obtain consent from users of virtual care apps. These agreements typically ask patients to consent to the retention, use, and disclosure of collected health information for purposes that are not clearly specified or which are unrelated to providing medical care. Putting aside issues with lay comprehension of the often verbose legalese in these agreements, consent is assumed from “clicking-through” regardless of whether the user actually read or understood the agreement. For example, Babylon’s Privacy Policy asks users to consent to sharing their health data with other companies and foreign governments. Under applicable federal and provincial legislation, health information can be used, retained or disclosed only for purposes authorized by patients, or, absent such authorization, for specified purposes.^15^ PIPEDA requires that organizations generally seek “express consent when the information is likely to be considered sensitive.” Given the sensitive nature of health information, authorization obtained for broad, unspecified use, disclosure, or retention of health information is likely to be legally problematic.

Ensuring data security is arguably the most challenging aspect of virtual care. With the explosion of communication technologies, there are many reliable and affordable options available to both physicians and patients. For virtual consultations with their own patients, doctors often rely on phones and popular, consumer friendly conferencing software such as Zoom, Skype, and Facetime.^18,19^ However, these software are notoriously insecure and vulnerable to security breaches. For example, Zoom and Skype have recently faced breaches, including hacking, cyberattacks, leaks of private information, and unconsented sharing of user data with advertisers.^20,21^ Privacy experts are also concerned with the encryption used by Zoom.^22^

Standalone platforms designed specifically for virtual medicine may attract less attention from cyberattackers, at least until they gain widespread use. It is presently unclear if medical apps are designed to withstand security attacks because these technologies were introduced without the completion of privacy impact assessments. Saskatchewan’s Information and Privacy Commissioner recently cautioned health professionals and patients to “be careful what you sign up for.”^23^ Alberta’s Commissioner similarly noted “concerns” with Babylon and launched an investigation into the app. She encouraged “physicians or patients with concerns about this app to remain opted out of using it,” while her office reviews its compliance with privacy laws.^24^

## Concerns with AI symptom checkers

Some virtual platforms include additional features, the most problematic of which use artificial intelligence (AI) technology to provide medical recommendations to users. The algorithms that produce these recommendations have not been subject to rigorous independent scrutiny and, given their proprietary nature, this oversight may never occur. There is also a lack of regulatory oversight over these technologies.^25,26^

Numerous concerns have already been reported with the accuracy of Babylon’s symptom-checker. For example, it recommended that patients seek emergency care for conditions that could be managed in the community (at a time when patients were avoiding unnecessary hospital visits), and according to a complaint to the UK’s medical device regulator, it misdiagnosed a heart attack as a panic attack.^27^ Although the app cautions that its recommendations should not be substituted for medical advice, individuals may not view recommendations with sufficient skepticism given the app’s promotion by government and the fact that consultations with actual doctors are integrated into the same platform. Although Babylon claims that its symptom checker performs better than doctors, Fraser et al. conclude that “Babylon’s study does not offer convincing evidence that its Babylon Diagnostic and Triage System can perform better than doctors in any realistic situation, and there is a possibility that it might perform significantly worse.”^28^

## Recommendations for policy-makers

Although virtual care was rapidly adopted in response to COVID-19, policy-makers now have an opportunity to reevaluate before it becomes entrenched as a permanent feature of the healthcare delivery system. We make three recommendations that will help mitigate the concerns with virtual care.

First, given the uncertain custodianship and ownership of the data generated under these models, the use of broad authorization for use and sharing of information, and data security issues, it is essential that policy-makers wait for privacy impact assessments before proceeding with virtual care so that it is implemented in a manner that complies with privacy laws. Although health providers adopted various ways of communicating electronically with their patients during COVID-19, governments and professional self-regulatory bodies must develop guidance on the permanent adoption of virtual care to ensure that they are meeting their obligations as custodians of health information.

Second, because of the convenience of virtual care (and the resulting incentive for some patients to prefer it over in-person consultations), policy-makers should prioritize virtual models that facilitate electronic access to one’s own doctor over virtual walk-in clinic doctors with whom patients do not have an established relationship. Given that continuity of care is linked to better patient outcomes, it is essential for governments to implement policies ensuring that all Canadians have access to a consistent primary care provider, rather than relying on virtual walk-in clinics as anyone’s main point of contact with the healthcare system. Policy-makers must also address the fact that virtual models will naturally exclude some groups, such as those without access to the requisite hardware and a reliable internet connection.

Third, policy-makers must ensure the public understands the significant limitations on AI-generated health recommendations. For example, they may consider not adopting virtual care models that integrate medical recommendations, given that patients may overestimate the reliability of recommendations that come from the same platform they use to consult with doctors. Health Canada must also ensure that its regulatory approach keeps pace with these emerging technologies.

## Conclusion

While virtual care can enhance access to health services, policy-makers must address significant concerns before forging ahead with its permanent implementation in order to preserve quality of care and protect privacy. With the public’s considerable appetite for virtual care^29^ and Prime Minister Trudeau’s recent announcement of $240.5 million to “develop, expand, and launch virtual care and mental health tools,”^30^ it seems virtual care is here to stay.
